# Studies on the anti-angiogenic effect of *Marsdenia tenacissima* extract *in vitro* and *in vivo*

**DOI:** 10.3892/ol.2013.1105

**Published:** 2013-01-04

**Authors:** ZHENGRONG HUANG, HAO LIN, YONG WANG, ZHIYUN CAO, WEI LIN, QIANG CHEN

**Affiliations:** 1 Department of Integrative Traditional Chinese and Western Medicine, Fujian Provincial Tumor Hospital, Fuzhou 350014;; 2 Fujian Academy of Integrative Medicine, Fujian University of Traditional Chinese Medicine, Fuzhou 350108;; 3 Department of Oncology, Union Hospital of Fujian Medical University, Fuzhou 350001, P.R. China

**Keywords:** *Marsdenia tenacissima*, angiogenesis, cancer treatment, herbal medicine

## Abstract

*Marsdenia tenacissima*, which is widely used as an anticancer herb in traditional Chinese medicine, has been shown to possess anticancer activities. However, the underlying molecular mechanism(s) involved in the anticancer effect of this herb are poorly understood. Angiogenesis is important in the development of cancer. The main features of angiogenesis are increased vasculature and overexpression of vascular endothelial growth factor (VEGF). In the present study, the effects of *M. tenacissima* extract (MTE) on human umbilical vein endothelial cell (HUVEC) proliferation, migration and capillary-like tube formation were investigated *in vitro* and using the chick embryo chorioallantoic membrane (CAM) assay *in vivo*. It was observed that MTE inhibited the proliferation of HUVECs by blocking the cell cycle progression from G1 to S. In addition, MTE inhibited the migration and tube formation of HUVECs. MTE treatment decreased the VEGF-A expression in human hepatoma cells (HepG2), as well as the expression of VEGF-A and VEGF receptor (VEGFR)-2 in HUVECs. MTE exposure in the CAM was able to reduce the formation of blood vessels in chick embryos. Overall, the present data suggest that extracts of *M. tenacissima* may serve as potential anti-angiogenesis agents.

## Introduction

Chemotherapy is one of the key tools used in the treatment of cancer, but drug resistance and severe adverse side-effects are significant obstacles (1–3). New drugs with improved features are urgently required to overcome drug resistance in various types of cancer. Natural products have been a source in the search for anticancer molecules and their further development into anticancer drugs. Traditional Chinese medicine is proving to be a promising resource for identifying drugs with anti-cancer activity (4,5). In recent years, interest in using natural products as therapeutic agents for cancer has increased.

*Marsdenia tenacissima*, the stem of *Marsdenia tenacissima* (Roxb.) *Wight et Arn*. (Family *Asclepiadaceae*), is grown widely in the southern provinces of China. It is used as a herbal medicine for the treatment of asthma, cancer, trachitis, tonsillitis, pharyngitis, cystitis and pneumonia (6,7). There are two major active constituents in *M. tenacissima*: phenolic acid and C21 steroidal glycosides (8). Preliminary clinical studies in China suggest that *M. tenacissima* is beneficial for treating patients with cancers such as esophageal cancer, gastric cancer and lung cancer, and has no significant side-effects (9,10). Extracts of *M. tenacissima* have been reported to have anticancer activity, inhibiting cancer growth and inducing apoptosis in several cancer cell lines *in vitro*(11–14). However, whether *M. tenacissima* inhibits tumor angiogenesis and its underlying mechanism(s) in endothelial cells remains unknown.

Angiogenesis is a crucial step for tumor growth and progression (15,16). It is a complex multistep process which includes the destabilization of established vessels, endothelial cell proliferation, migration and tubulogenesis (17). Tumor angiogenesis is a process wherein a network of blood vessels penetrates a cancerous growth to supply nutrients and oxygen and remove metabolic waste from the tumor. Tumor growth and metastasis depends heavily on angiogenesis. The induction of angiogenesis is mediated by a variety of molecules secreted from the cells within the tumors. It is well known that vascular endothelial growth factor (VEGF) is crucial in regulating angiogenesis (18) and has become a key focus of antiangiogenic therapy.

In the present study, the effect of *M. tenacissima* extract (MTE) on the antiangiogenic response was studied using *in vitro* and *in vivo* angiogenesis models. Furthermore, the possibility of MTE reacting with the vascular endothelial cells specifically through VEGF receptors was also investigated.

## Materials and methods

### Reagents

MTE was provided by Nanjing Sanhome pharmeceutical Co. Ltd. (Nanjing, China). RPMI-1640 medium, Dulbecco’s modified Eagle’s medium (DMEM), fetal bovine serum (FBS), penicillin-streptomycin, trypsin-EDTA and TRIzol reagent were purchased from Invitrogen (Carlsbad, CA, USA). The cell cycle assay kit was purchased from BD Biosciences (San Jose, CA, USA), the SuperScript II reverse transcriptase kit from Promega (Madison, WI, USA) and the *in vitro* angiogenesis assay kit from Millipore (Billerica, MA, USA). Human VEGF-A and VEGF receptor-2 (VEGFR-2; KDR) ELISAs were obtained from R&D Systems (Minneapolis, MN, USA). All other chemicals were purchased from Sigma Chemicals (St. Louis, MO, USA).

### Cell culture

Human umbilical vein endothelial (HUVECs) and human hepatoma cells (HepG2) were obtained from the American Type Culture Collection (ATCC, Manassas, VA, USA). HUVECs and HepG2 cells were grown in RPMI-1640 and DMEM, respectively, supplemented with 10% (v/v) FBS, 100 U/ml penicillin and 100 *μ*g/ml streptomycin and incubated at 5% CO_2_ in a 37°C incubator.

The study was approved by the Ethics Committee of Fujian University of Traditional Chinese Medicine, Fuzhou, China.

### Cell viability assay

Cell viability was evaluated using the 3-(4,5-dimethylthiazol-2-yl)-2, 5-diphenyl tetrazolium bromide (MTT) colorimetric assay. HUVECs were seeded into 96-well plates at 1×10^4^ cells/well. The cells were treated with 0, 2.5, 5 and 7.5 mg/ml of MTE for 24 h. At the end of the treatment, 20 *μ*l of the MTT (5 mg/ml) was added to each well. After 4 h, MTT was removed and 100 *μ*l of DMSO was added to each well. The absorbance was measured at 490 nm with a microplate reader (BioTek, Winooski, VT, USA). The cell viability was calculated as follows: cell viability (%) = (average absorbance of MTE-containing serum group / average absorbance of blank group) × 100.

### Evaluation of cell confluency

HUVECs were seeded at a concentration of 2×10^5^ cells/well into a 6-well plate. The cells were treated with 0, 2.5, 5 and 7.5 mg/ml of MTE for 24 h. Cell confluency was evaluated using a phase-contrast microscope at a magnification of ×200 (Olympus, Tokyo, Japan).

### Cell cycle analysis

After incubating the HUVECs with various concentrations of MTE for 24 h, the cells were harvested and adjusted to a concentration of 1×10^6^ cells/ml. Cell cycle analysis was evaluated using flow cytometry and the percentages of G0/G1-phase, S-phase and G2/M-phase were calculated with the ModFit software (BD Biosciences).

### Wound-healing assay

HUVECs were seeded at a concentration of 2×10^5^ cells/well, into a 12-well plate. Following 24 h of incubation, cells were scraped away vertically in each well using a pipette tip. Three randomly selected views along the scraped line were photographed on each well using a phase-contrast inverted microscope at ×100 magnification. The cells were further treated with various concentrations of MTE (0, 2.5, 5, 7.5 mg/ml) for 24 h and another set of images was recorded. A reduction in the scraped area was considered to be the indicative of wound-healing and cell migration.

### Capillary-like tube formation assay

Tube formation by HUVECs was evaluated using the *in vitro* angiogenesis assay kit, according to the manufacturer’s instructions. After the HUVECs were treated with MTE, cells were harvested and diluted to 1×10^4^ cells in 50 *μ*l medium. The cells were then seeded onto a solid gel of basement proteins (ECMatrix gel) in 12-well plates and incubated for 9 h at 37°C. Cellular morphology and the development of capillary tube networks were evaluated using a phase-contrast inverted microscope. Images were obtained at a magnification of ×100.

### VEGF-A and VEGFR-2 RT-PCR analysis

HUVECs or HepG2 cells were seeded at a concentration of 2×10^5^ cells into a 6-well plate and treated with various concentrations of MTE for 24 h. Total RNA was isolated with TRIzol reagent (Invitrogen). Oligo(dT)-primed RNA (1 *μ*g) was reverse-transcribed with SuperScript II reverse transcriptase and the cDNA was used to determine the amount of VEGF-A or VEGFR-2 mRNA by PCR with Taq DNA polymerase (Fermentas, Waltham, MA, USA) using the VEGF-A (S: GCC TTG CCT TGC TGC TCT A, AntiS: GAT GTC CAC CAG GG TCT CG), VEGFR-2 (S: ACG CCG ATT ATG TGA GA, AntiS: AGGC AGG AGT TGA GTA TGT) and GAPDH (S: GTC ATC CAT GAC AAC TTT GG, AntiS: GAG CTT GAC AAA GTG GTC GT) primers.

### VEGF-A and VEGFR-2 ELISA assay

HUVECs and HepG2 cells were seeded at a concentration of 2×10^5^ cells into a 6-well plate and treated with various concentrations of MTE for 24 h. Cells were collected to measure the secretion level of VEGF-A in the two cell lines and cell lysates were used to determine the protein expression levels of VEGFR-2 in the HUVECs. The measurements were performed using the Quantikine ELISA kit, according to the manufacturer’s instructions.

### Chick chorioallantoic membrane (CAM) assay

A CAM assay was performed to determine the *in vivo* anti-angiogenic activity of MTE. MTE (1 mg) was loaded on to 0.5 cm-diameter Whatman filter paper and then applied to the CAM of a seven-day-old embryo. Following incubation for 72 h at 37°C, the angiogenesis around the filter was recorded. The number of blood vessels in a circular perimeter surrounding the implants, at a distance of 0.25 cm from the edge of the filter was counted manually.

### Statistical analysis

All data are the mean of three replicates, with the exception of the CAM assays in which 10 replicates were performed for each data point. The data were analyzed using the SPSS software (Version 11.5). Statistical analysis of the data was performed using the Student’s t-test and analysis of variance (ANOVA). P<0.05 was considered to indicate statistically significant differences.

## Results

### MTE inhibits the proliferation of HUVECs

**Figure 1:**
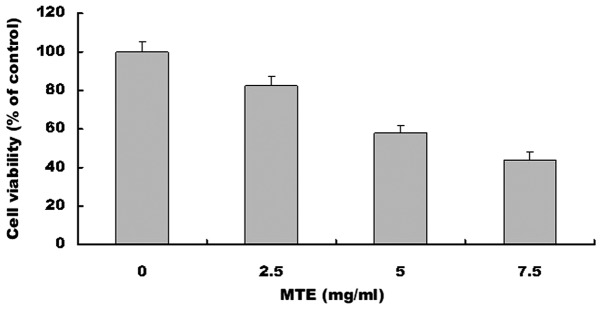
Effect of MTE on HUVEC viability. The cells were treated with 0, 2.5, 5 and 7.5 mg/ml of MTE for 24 h. The cell viability was determined by the MTT assay. The data were normalized to the viability of the control cells (100%, treated with 0.5% DMSO as vehicle). Data are the mean ± SD (error bars) from three independent experiments. ^*^P<0.01, vs. control cells. MTE, *Marsdenia tenacissima* extract; HUVEC, human umbilical vein endothelial cell; MTT, 3-(4,5-dimethylthiazol-2-yl)-2, 5-diphenyl tetrazolium bromide.

**Figure 2:**
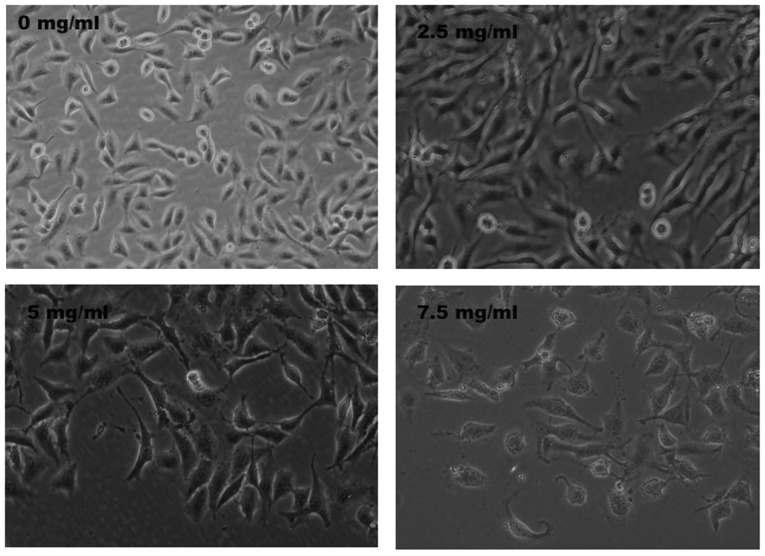
Effect of MTE on HUVEC confluency. Cells were treated with 0, 2.5, 5 and 7.5 mg/ml of MTE for 24 h and the changes were observed using phase-contrast microscopy. The images were obtained at a magnification of ×200. Images are a representative of three independent experiments. MTE, *Marsdenia tenacissima* extract; HUVEC, human umbilical vein endothelial cell.

HUVEC viability was determined following treatment with various concentrations of MTE for 24 h. Treatment with 2.5 to 7.5 mg/ml of MTE for 24 h dose dependently reduced the cell viability from 56 to 17%, when compared with the control cells (P<0.01; Fig. 1). To further verify these results, the effect of MTE on HUVEC confluency was observed via phase-contrast microscopy. MTE treatment led to a gradual decrease in the confluency of the monolayer with the increase in drug concentration (Fig. 2).

### MTE blocks cell cycle progression in HUVECs

**Figure 3:**
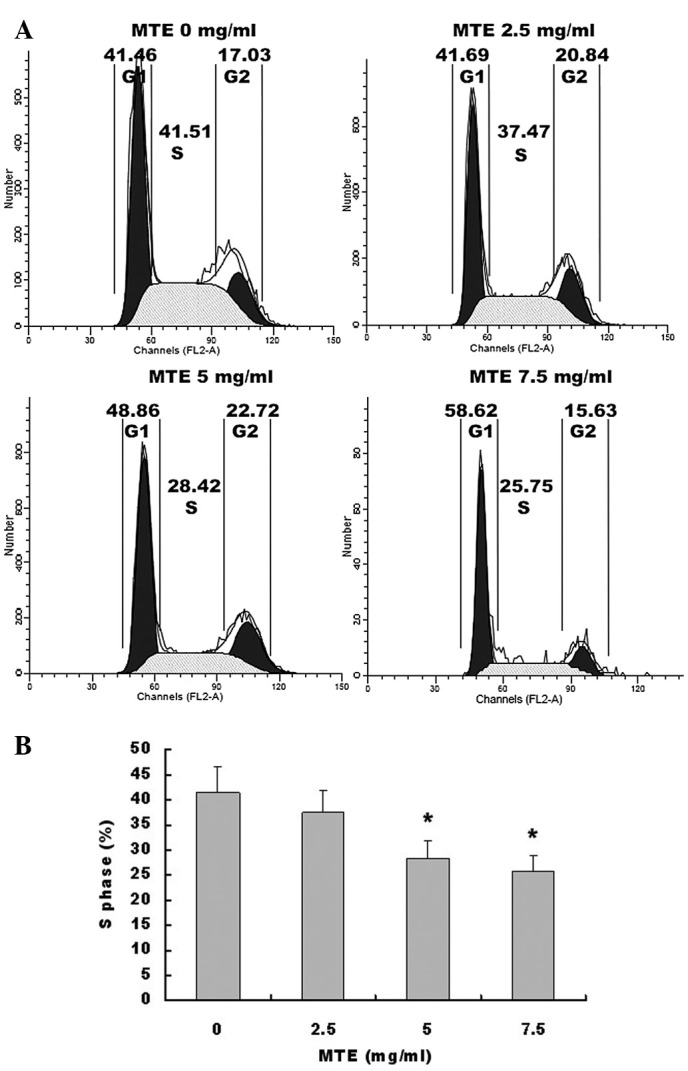
Effect of MTE on HUVEC cell cycle progression. (A) The cells were treated with 0, 2.5, 5 and 7.5 mg/ml of MTE for 24 h, stained with PI and analyzed by FACS. The proportion of cells in each phase of the cell cycle was calculated using ModfitLT Version 3.0 Software. Representative assays are shown for each concentration of MTE. (B) The percentages of cells in the S phase, following the treatment with 0, 2.5, 5 and 7.5 mg/ml of MTE were compared. Data shown are the mean ± SD (error bars) from three independent experiments. ^*^P<0.01, vs. control cells. MTE, *Marsdenia tenacissima* extract; HUVEC, human umbilical vein endothelial cell; PI, propidium iodide.

To test whether the treatment of cells with MTE was able to cause cell cycle arrest, the cell cycle distribution was analyzed by flow cytometry following the treatment of the HUVECs with 0, 2.5, 5 and 7.5 mg/ml of MTE for 24 h. As shown in Fig. 3A and B, the percentage proportions of S phase cells following treatment with 0, 2.5, 5 and 7.5 mg/ml of MTE were 41.51±5.2, 37.47±4.5, 28.42±3.5 and 25.75±3.2%, respectively (P<0.01), suggesting that MTE inhibits HUVEC proliferation by blocking the cell cycle progression from G1 to S.

### MTE inhibits HUVEC migration and tubulogenesis

**Figure 4:**
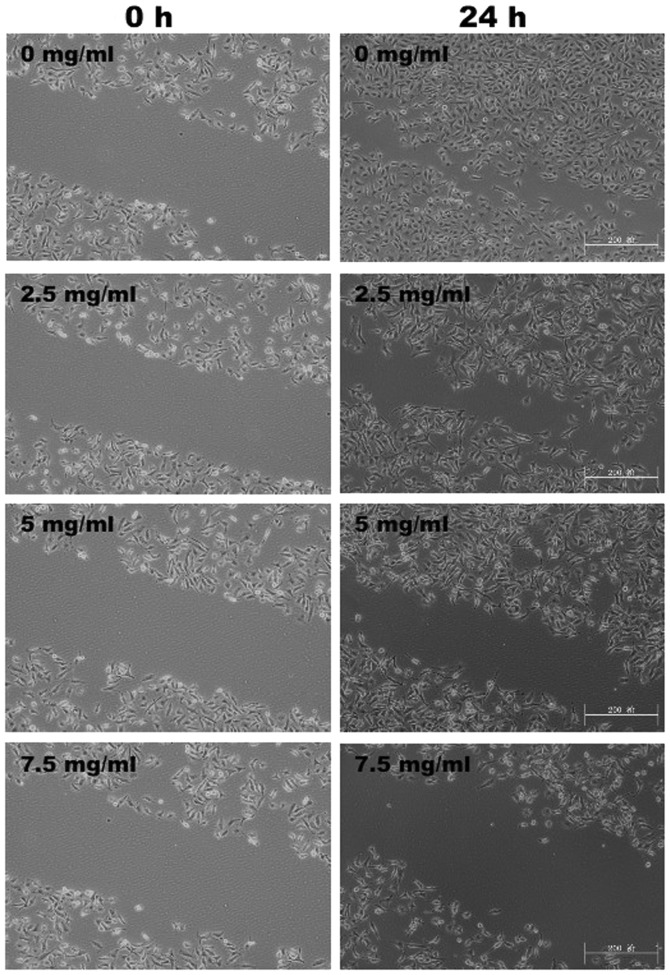
Effect of MTE on HUVEC migration. After treatment with the indicated concentrations of MTE for 24 h, the migration pattern of the HUVECs was observed using phase-contrast microscopy. The images were obtained at a magnification of ×100. Images are representative of three independent experiments. MTE, *Marsdenia tenacissima* extract; HUVEC, human umbilical vein endothelial cell.

**Figure 5:**
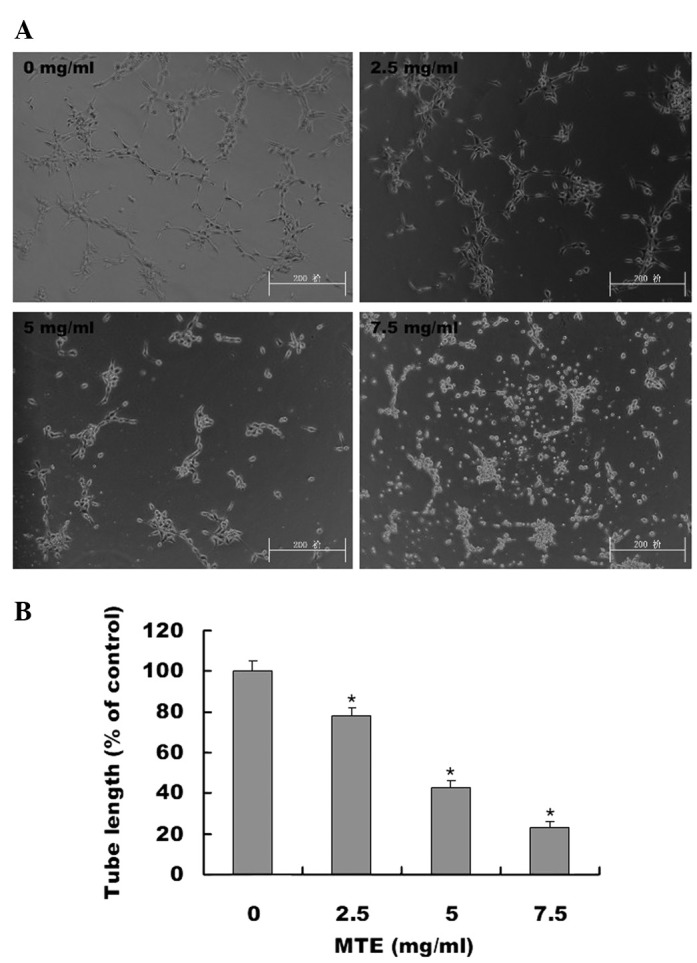
The effect of MTE on tube formation in HUVECs. (A) HUVECs were harvested and suspended in medium containing various concentrations of MTE. The harvested cells were then seeded in basement protein (ECMatrix, Millipore)-coated plates and incubated for 9 h at 37°C. The development of network-like, tube structures was observed by phase-contrast microscopy. The images were obtained at a magnification of ×100. Images are representative of three independent experiments. (B) The total length of the capillary-like tubes was measured and normalized to the control. Data shown are the mean ± SD (error bars) from three independent experiments. ^*^P<0.01, vs. control cells. MTE, *Marsdenia tenacissima* extract; HUVEC, human umbilical vein endothelial cell.

To assess the antiangiogenic properties of MTE *in vitro*, its inhibitory effects on the chemotactic motility of HUVECs were investigated using the wound-healing migration assay. As shown in Fig. 4, 24 h post-wounding, untreated HUVECs migrated into the wounded (clear) area of the cell monolayer, whereas MTE treatment markedly inhibited the HUVEC migration in a dose-dependent manner. Furthermore, how MTE regulates the capillary tubule formation of HUVECs was studied. As shown in Fig. 5A and B, untreated HUVECs formed elongated tube-like structures. By contrast, MTE treatment resulted in a significant decrease in capillary tube formation in a dose-dependent manner.

### MTE suppresses the expression of VEGF-A and VEGFR-2

**Figure 6:**
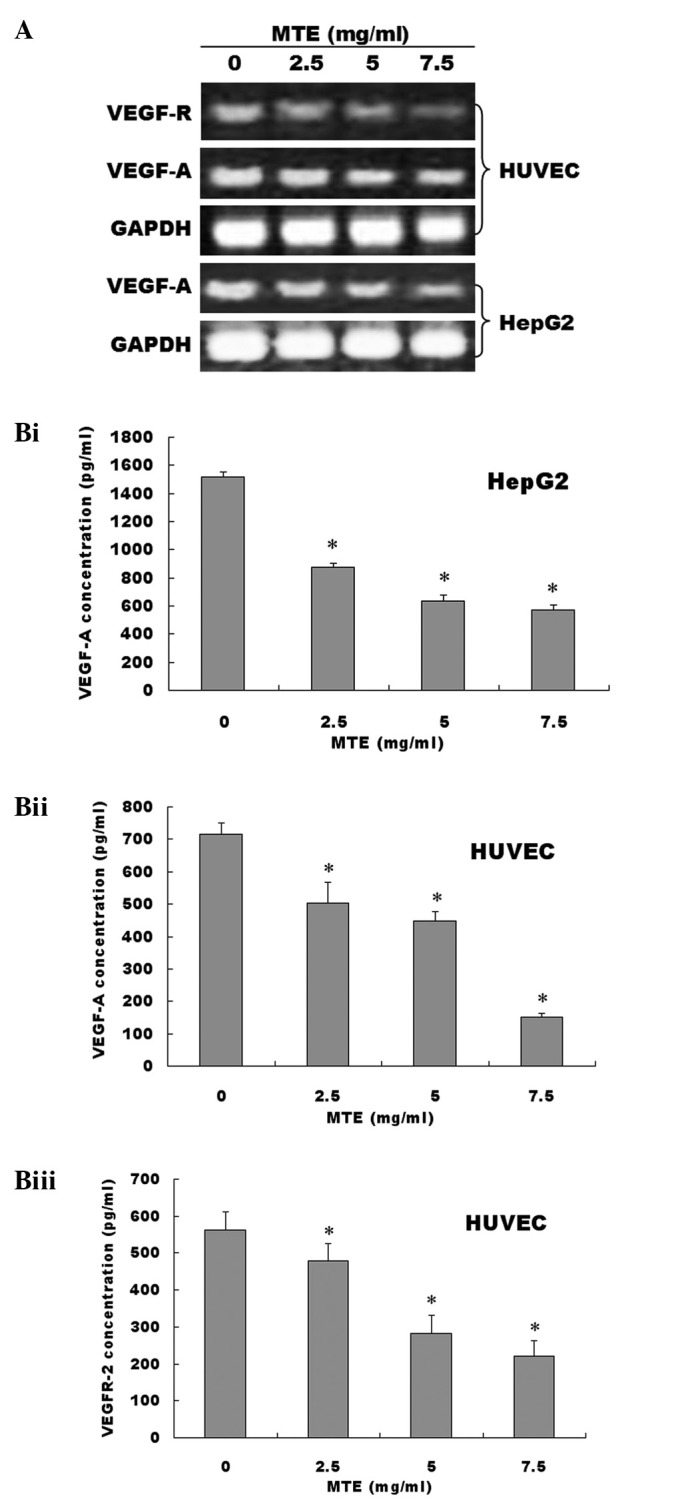
(A) Effect of MTE on the mRNA expression levels of VEGF-A and VEGFR-2. Cells were treated with various concentrations of MTE for 24 h. The mRNA levels of VEGF-A and VEGFR-2 were determined by RT-PCR in HUVECs and HepG2s. GAPDH was used as an internal control. Data are representative of three independent experiments. (B) Effect of MTE on the protein expression levels of VEGF-A and VEGFR-2.(Bi) The protein levels of VEGF-A secreted in cell culture medium were determined by ELISA after HepG2 cells were treated with indicated concentrations of MTE for 24 h. (Bii) The protein levels of VEGF-A secreted in cell culture medium were determined by ELISA after HUVECs cells were treated with indicated concentrations of MTE for 24 h.. (Biii) The protein levels of VEGFR-2 in cell lysates were determined by ELISA after HUVECs cells were treated with indicated concentrations of MTE for 24 h. Data are the mean ± SD (error bars) from at least three independent experiments. ^*^P<0.01, vs. control cells. MTE, *Marsdenia tenacissima* extract; VEGF-A, vascular endothelial growth factor-A; VEGFR-2, vascular endothelial growth factor receptor-2; HUVEC, human umbilical vein endothelial cell; HepG2, human hepatoma cell.

To further investigate the underlying mechanism of MTE’s anti-angiogenic activity, the effects of MTE on VEGF-A expression and secretion in HUVECs and HepG2 cells and the expression of VEGFR-2 in HUVECs were investigated. The results of the RT-PCR assay showed that MTE treatment reduced VEGF-A mRNA expression in HepG2 cells and HUVECs in a dose-dependent manner, as well as suppressing VEGFR-2 mRNA expression in HUVECs (Fig. 6A). Moreover, the protein expression patterns of VEGF-A and VEGFR-2 showed similar changes, as the mRNA levels decreased according to the MTE treatment (Fig. 6B).

### MTE inhibits angiogenesis in vivo

**Figure 7:**
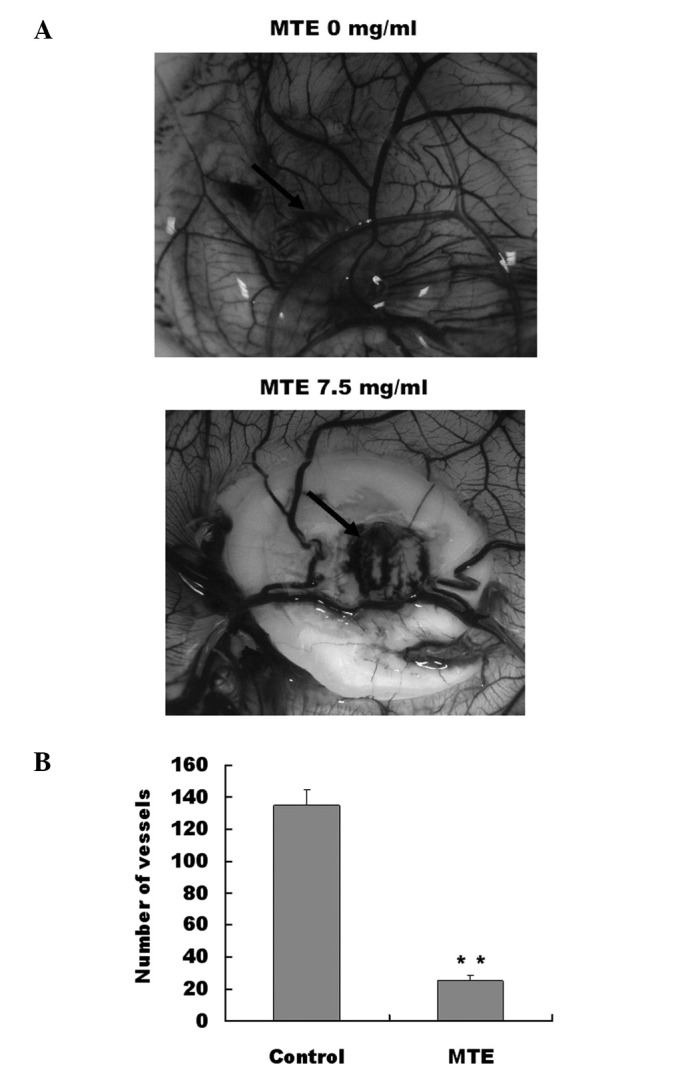
Effect of MTE on angiogenesis *in vivo*. (A) A 0.5 cm-diameter filter paper loaded with 1.0 mg of MTE was applied to the chick CAM and incubated at 37°C for 72 h. The blood vessels surrounding the filter were photographed. Images are representative images of the results obtained from the two groups. Arrow: filter paper. (B) The number of blood vessels was quantified manually in a circular perimeter 0.25 cm from the edge of the filter paper inserts. Assays were performed twice with a total of 10 eggs for each tested concentration of MTE. Data are the mean ± SD (error bars). ^*^P<0.01, vs. control. MTE, *Marsdenia tenacissima* extract; CAM, chorioallantoic membrane.

For tumors to grow they must be able to integrate successfully with normal host tissues at the primary tumor site and metastastic sites. Tumors require a blood supply to sustain growth. Therefore, the effect of MTE on microvessel formation *in vivo* was studied in the CAM model. The number of vessel branch points was determined in the absence and presence of MTE. As shown in Fig. 7A and B, MTE treatment significantly reduced the total number of blood vessels in the chicken embryos when compared with the untreated control. This result provided evidence that the anti-angiogenic effects of MTE may be important in its anti-cancer activity.

## Discussion

The present study demonstrates that MTE is highly effective at inhibiting cell proliferation in HUVECs by blocking the cell cycle progression from G1 to S. In addition, the present study showed that MTE inhibits endothelial cell migration and prevents vascular formation *in vitro*. Collectively, the present data suggest that MTE affects angiogenesis by targeting endothelial cells directly. MTE was also able to suppress the growth of blood vessels in CAM models *in vivo*.

Angiogenesis is important in providing nutrients and oxygen to the growing tumor (19). The process of cancer-associated angiogenesis is highly regulated, involving several growth factors and cytokines. Studies suggest that tumor growth and dissemination are dependent on the development of a neovasculature and that VEGF is a primary stimulator of angiogenesis (20). VEGF regulates vascular formation through the initiation of vessel growth, incorporation of hematopoietic and endothelial progenitor cells into the developing vasculature and inhibition of endothelial cell apoptosis (21). VEGF exerts its biological effects primarily through interaction with its specific receptors, VEGFRs (22,23). Although VEGF is a ligand for VEGFR-1 and VEGFR-2, VEGF signaling in angiogenesis is mainly mediated through VEGFR-2, a receptor tyrosine kinase expressed at elevated levels in endothelial and cancerous cells (24). Inhibition of VEGF/VEGFR is a key strategy in anti-angiogenic therapy for cancer. The present study showed that MTE inhibited VEGF-A and VEGFR-2 expression in HUVECs. The selective inhibition of angiogenic factors suggests that MTE has an anti-angiogenic effect, specifically through VEGF suppression. However, this requires further study, to understand whether MTE inhibits VEGF expression through other pathways.

In the present study, MTE inhibited angiogenesis *in vivo* and suppressed the key steps involved in angiogenesis, including proliferation, survival, migration and tubulogenesis in endothelial cells. To the authors’ knowledge, this is the first study demonstrating the expression of VEGF/VEGFR in HUVECs in response to MTE.

In conclusion, the present study demonstrated for the first time that MTE is able to inhibit tumor angiogenesis *in vitro* and *in vivo* by targeting VEGF/VEGFR in the signal pathway. The present observation of the anticancer effects of *M. tenacissima* not only supports its ethnopharmacological value, but also confirms its potential as a new anti-angiogenesis agent.
